# Ankle-Preserving Surgery for Ankle Instability After Triple Arthrodesis: A Case Report

**DOI:** 10.7759/cureus.77702

**Published:** 2025-01-20

**Authors:** Nori Yamashita, Tadashi Kimura, Mitsuru Saito, Makoto Kubota

**Affiliations:** 1 Orthopaedic Surgery, Jikei University School of Medicine, Tokyo, JPN

**Keywords:** ball-and-socket joint, distal tibial oblique osteotomy, lateral displacement calcaneal osteotomy, revision for malalignment, triple arthrodesis, varus aligned feet

## Abstract

This report describes a case in which ankle-preserving surgery was performed for secondary ankle instability with good results in a patient with residual deformity after triple arthrodesis. A 42-year-old woman had undergone triple arthrodesis at the age of 16 years to correct a club foot deformity that had caused paralysis of the lower extremities. However, her left ankle instability worsened over time. Eventually, she visited our hospital, where she was found to have ankle instability and club foot. The ankle joint appeared to have a ball-and-socket shape, and plain radiographs showed a talus varus. The cause of the instability was varus deformity of the calcaneus in the standing position, which remained after the previous surgery. We performed a distal tibial oblique osteotomy and lateral displacement calcaneal osteotomy to improve ankle eversion. She could mobilize soon after surgery, reaching full load capacity at 12 weeks. After two years, her range of motion at the ankle and load-bearing stability were maintained, significantly improving her walking endurance. Triple arthrodesis is a common treatment for conditions associated with paralysis of the lower extremities. However, persistent malalignment can lead to prolonged ankle instability and deformity, prompting a need for additional procedures such as Dwyer surgery and ankle arthrodesis. In this case, although ankle joint instability was present, the range of motion was maintained without worsening arthropathy. Therefore, joint-preserving surgery was chosen. Notably, a literature search did not reveal any similar cases. Surgery to correct joint morphology in a patient with ankle instability improved ground contact and avoided varus deformity, resulting in positive outcomes.

## Introduction

Triple arthrodesis is a commonly performed surgical procedure for addressing advanced paralytic foot deformities. While effective in stabilizing the hindfoot, postoperative complications, such as progressive deformity of the ankle and foot, may occur. In such instances, corrective procedures, including ankle arthrodesis and calcaneal osteotomy, are often employed as effective strategies to address hindfoot malalignment [1]. However, despite the clinical importance of these corrective approaches, detailed studies and comprehensive reviews on this topic remain scarce.

In this report, we present a case involving a patient who, despite residual deformity following triple arthrodesis, demonstrated sufficient ankle range of motion and no evidence of progressive arthropathy. To address secondary ankle instability caused by the deformity, an ankle-preserving surgical approach was undertaken, yielding favorable clinical outcomes. This case underscores the feasibility and potential benefits of joint-preserving surgical strategies in managing residual deformities after triple arthrodesis.

## Case presentation

A 42-year-old woman presented with instability in her left ankle. Shortly after birth, she had undergone surgery for a spinal neuroblastoma causing paralysis below L5. During childhood, she underwent Achilles tendon lengthening and soft tissue release for club foot, and at the age of 16 years underwent triple arthrodesis for a foot deformity. However, she gradually developed ankle joint instability and an unstable gait. At the age of 40 years, she underwent Dwyer calcaneal osteotomy and distal tibial oblique osteotomy on the right side at another hospital but developed a deep infection and required early removal of the plate, which made further treatment difficult. She was then referred to our hospital.

**Figure 1:**
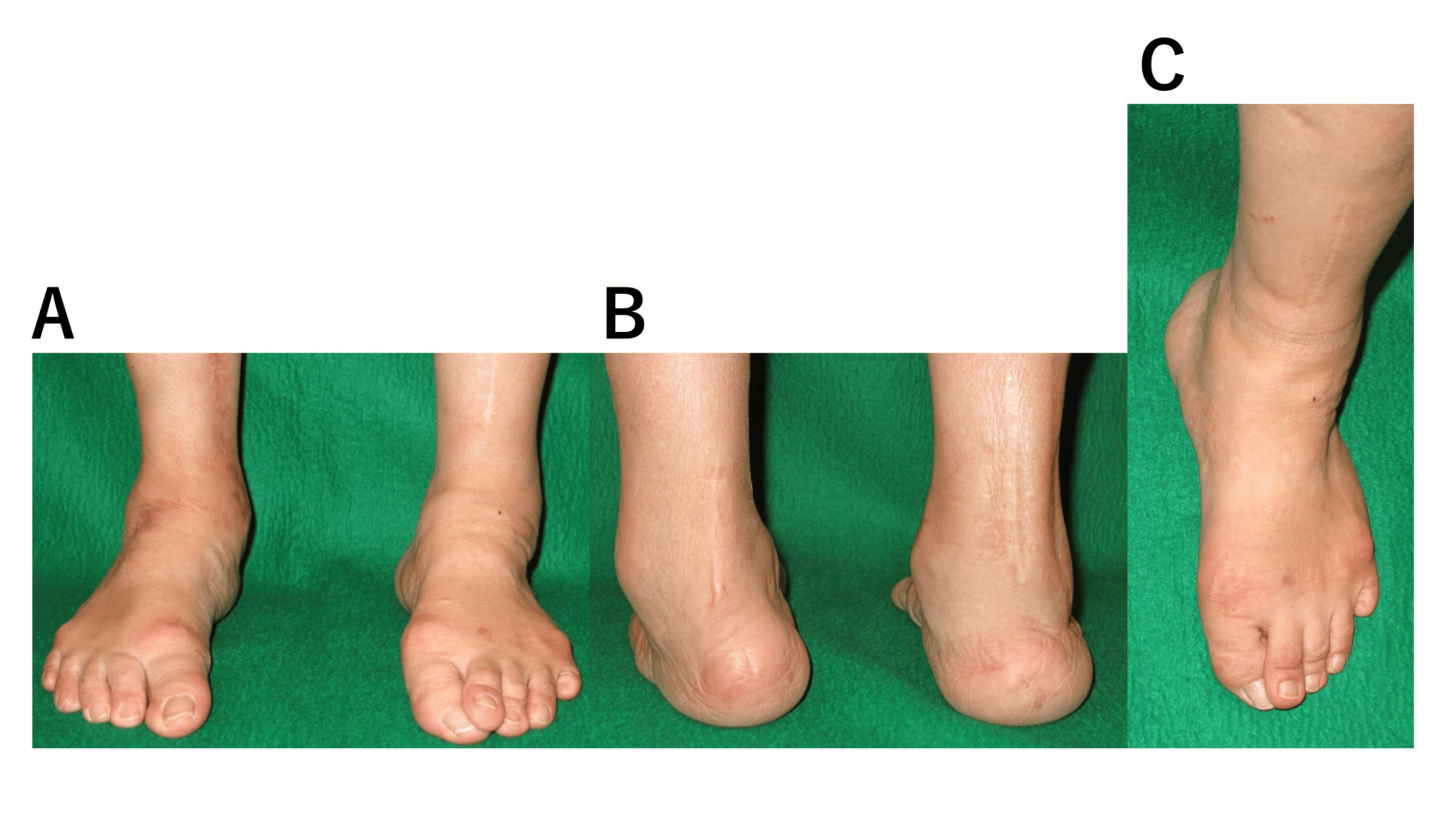
Clinical photographs obtained at the first visit. Front (A) and rear (B) views of both feet. In the left foot, the calcaneus was varus, the forefoot was supinated, and slight pes cavus was observed. (C) In the standing position, the second toe was over the first toe in the left foot.

At the first visit, she presented with calcaneus varus of the left foot, supination of the forefoot, and mild pes cavus but no pes equinus. In the standing position, the ball of the big toe did not contact the ground adequately owing to supination, and the second toe was over the first toe. Her passive range of motion at the left ankle joint was 10º of dorsiflexion and 40º of plantar flexion. However, she was hardly able to perform active movements because of the paralysis. She had sensory disturbance below L5, but her complaints of pain were mild. Her ankle joint instability meant that the lower limbs provided limited support, and continuous walking was limited to 300 m (Figure 1).

**Figure 2:**
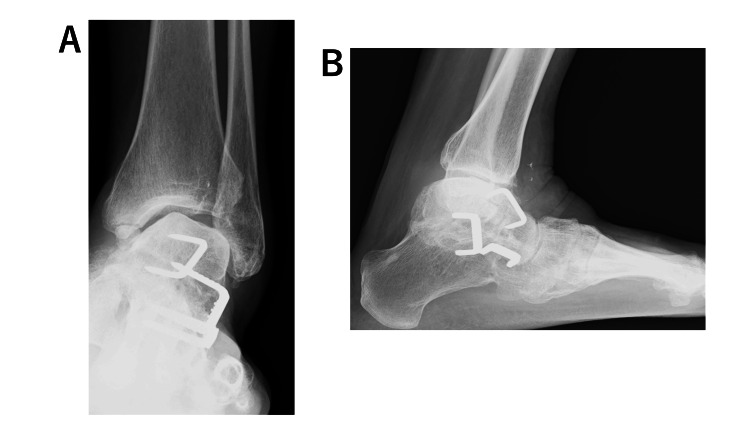
Plain radiographs of the left foot obtained at the first visit. (A) After triple arthrodesis, the plafond was tilted laterally downwards relative to the axis of the tibia, and there was varus displacement of the talus in the ankle mortise. (B) Lateral view showing flattening of the trochlea of the talus.

**Figure 3:**
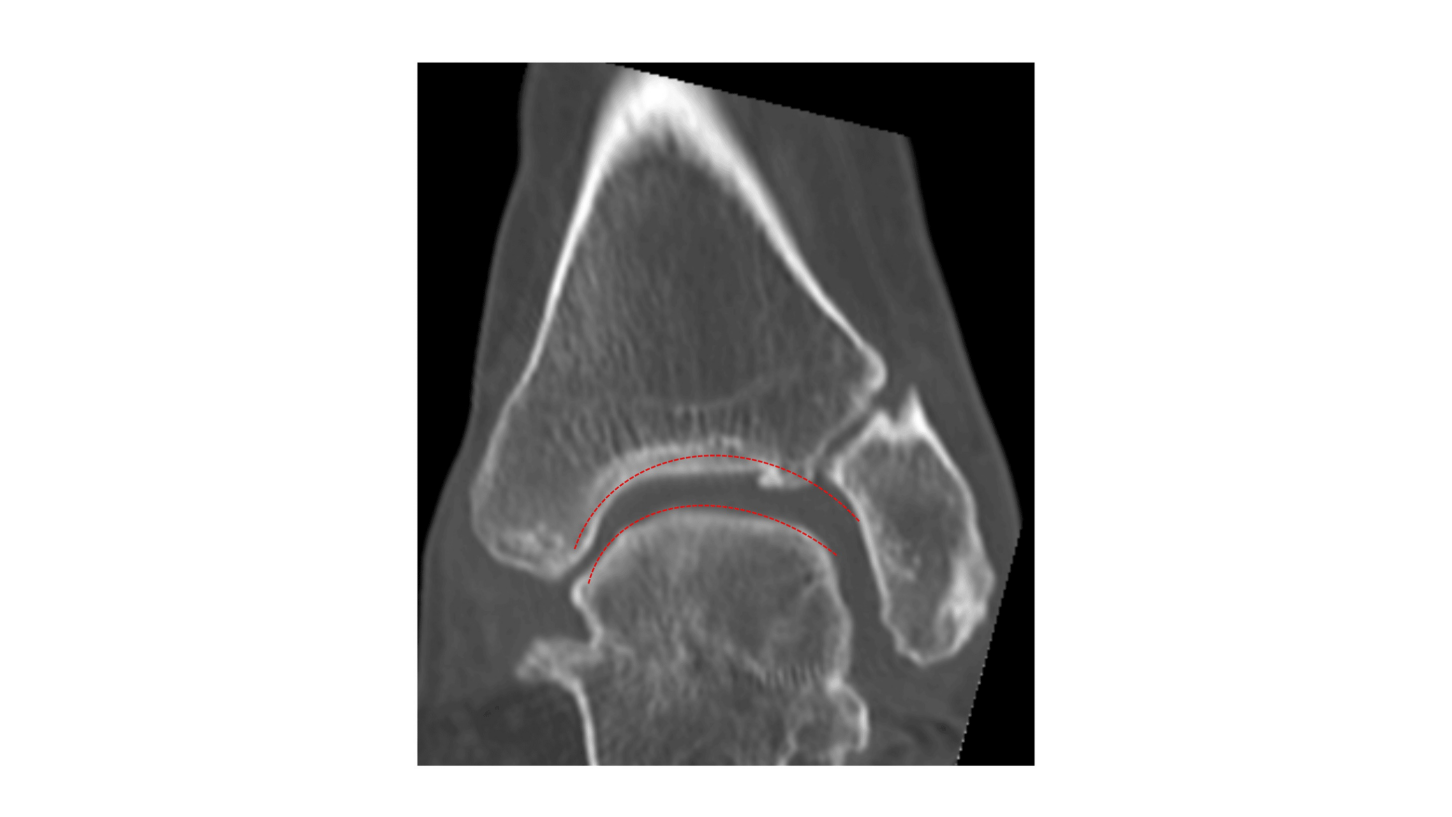
Computed tomography scan of the left foot showing a rounded talar margin and a ball-and-socket joint.

A lateral plain radiograph showed that the trochlea of the talus was flattened after the triple arthrodesis, the plafond was tilted laterally downwards relative to the axis of the tibia, and there was varus displacement of the talus in the ankle mortise. She also had mild pes cavus and an ankle deformity. The calcaneal pitch angle was 18º, the Meary’s angle was −22º, the tibial articular surface angle was 77º, and the talar tilt angle was 11º. A computed tomography scan revealed a rounded talar margin and a ball-and-socket joint (Figures 2, 3).

**Figure 4:**
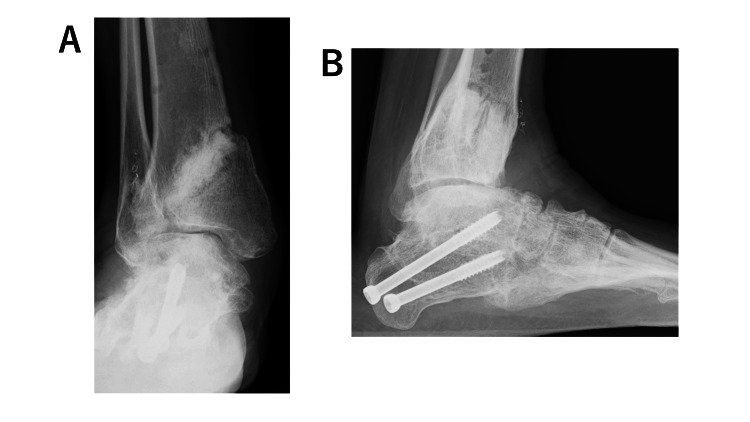
Plain radiographs of the right foot after the previous surgery. (A, B) The foot is plantigrade

Problems in this case included a club foot, instability of the ankle joint, and tilting of the plafond. Initially, ankle arthrodesis was considered. However, in the right foot, the varus of the calcaneus had resolved, the foot became plantigrade, and good support was obtained after previous surgery at another center. Therefore, we decided to perform the same joint-preserving surgery for the left ankle (Figure 4).

Surgical procedure

**Figure 5:**
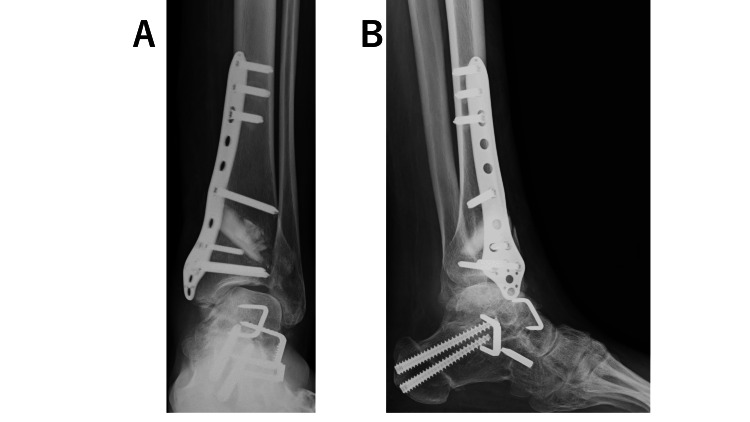
Plain radiographs of the left foot after the revision surgery. (A, B) Range of motion at the ankle joint was maintained and the stability was good.

The surgery was performed in the supine position under general anesthesia. First, lateral displacement calcaneal osteotomy was performed. An oblique skin incision of approximately 5 cm was made on the outside of the calcaneal body for entry to the joint. An osteotomy was performed to move the posterior part of the calcaneus 8 mm to the outside. Two headless screws with a maximum diameter of 6.5 mm (Acutrak Plus® Acumed, Hillsboro, OR, USA) were then used for fixation. Next, a distal tibial oblique osteotomy was performed. A longitudinal incision of approximately 6 cm was made in the medial proximal part of the ankle joint. The tibia was cut 4 cm from the center of the tip of the medial malleolus towards its outer and lower sides, and after advancing from the ankle joint 10 mm to the center and from the distal tibiofibular joint 10 mm to the medial site, an open wedge osteotomy was performed. When the medial side was dilated, the instability of the talus disappeared and its angle widened by 15º. After filling the gap with β-tricalcium phosphate, a MODE Distal tibial plate® (Teijin Nakashima Medical Co., Ltd., Okayama, Japan) was bent and fixed using a plate with a reduced curvature. After confirmation that the range of motion at the ankle joint was maintained and stability was good, the surgery was completed (Figure 5).

Postoperative course

**Figure 6:**
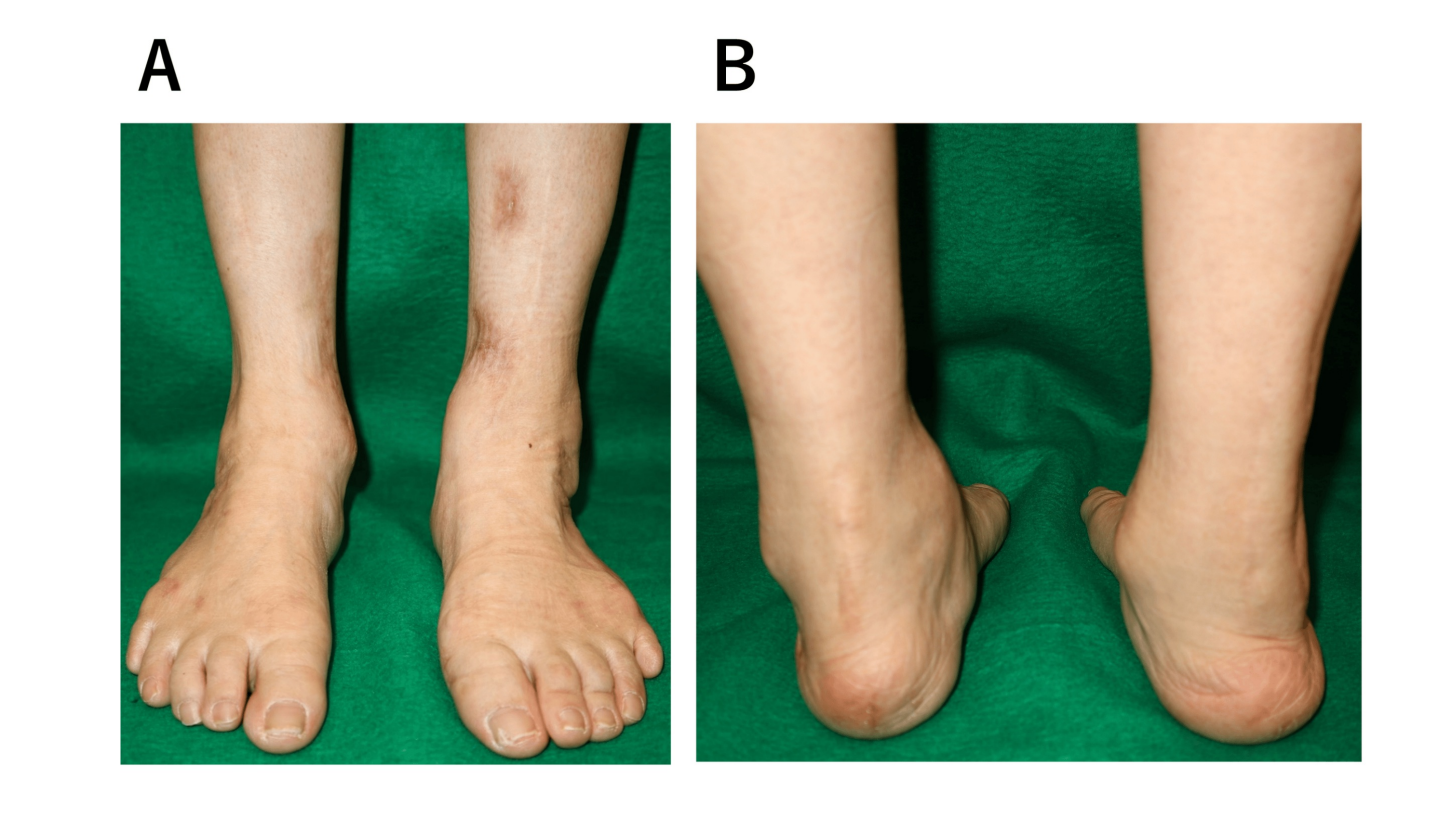
Clinical photographs of both feet obtained 4 years after the revision surgery. (A, B) The soles of both feet were plantigrade.

**Figure 7:**
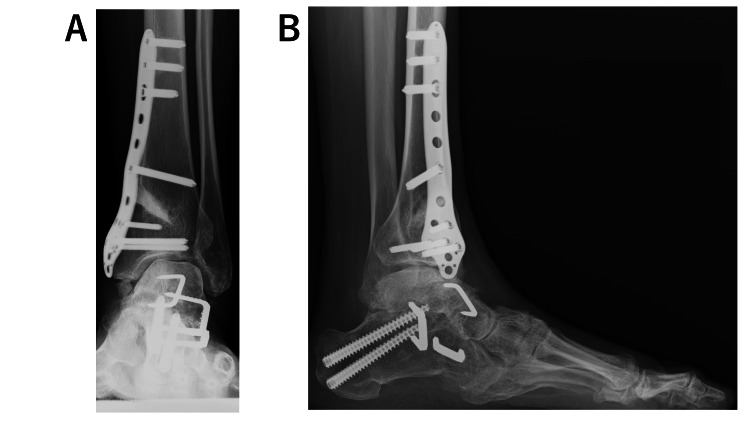
Plain radiographs of the left foot obtained 4 years after the revision surgery. (A, B) Some residual β-tricalcium phosphate can be seen remained in the in the frontal view, but bone fusion was complete.

**Figure 8:**
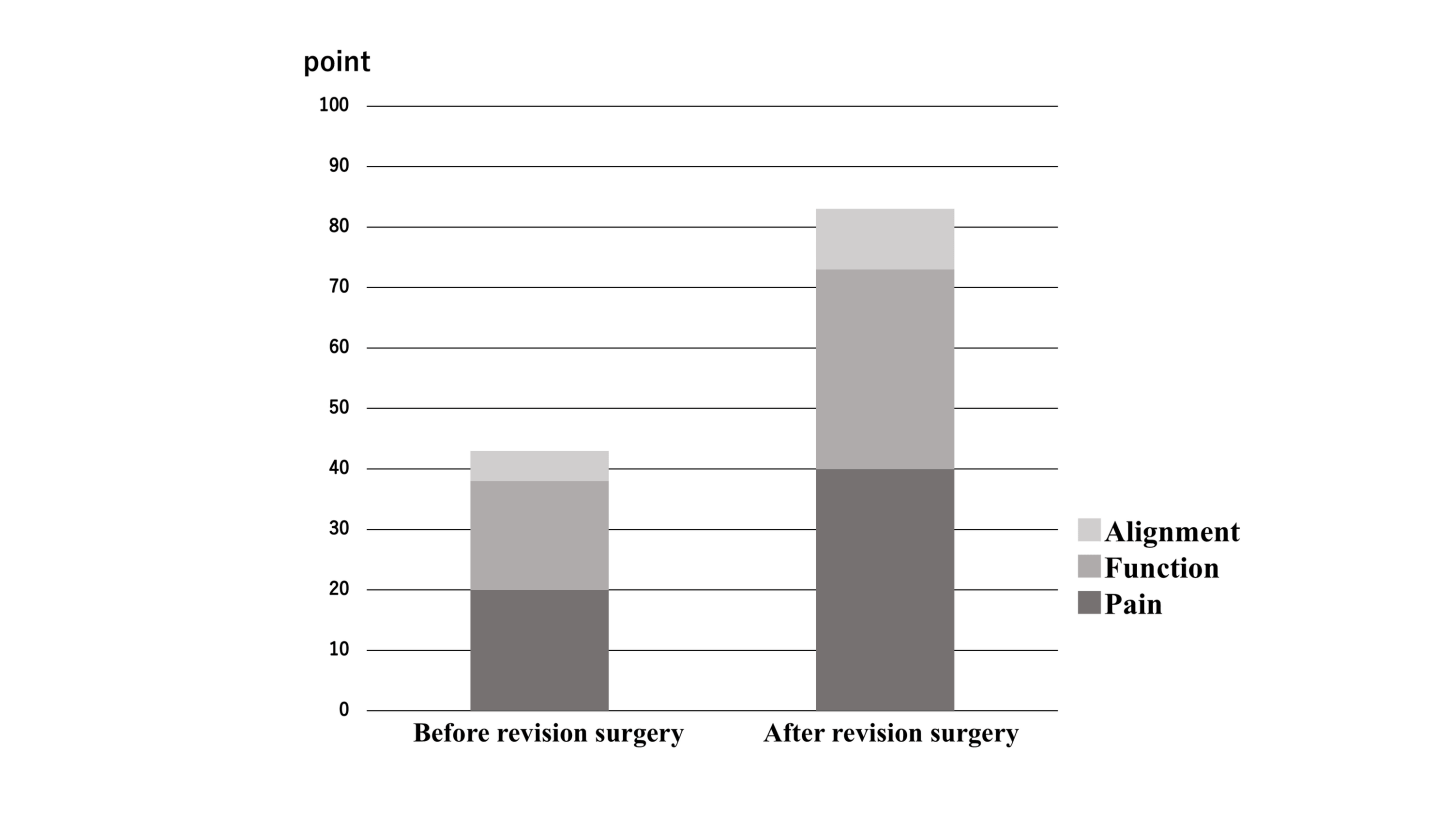
The JSSF score improved from 43 points before surgery to 88 points at 4 years after the revision surgery. JSSF: Japanese Society for Surgery of the Foot

**Figure 9:**
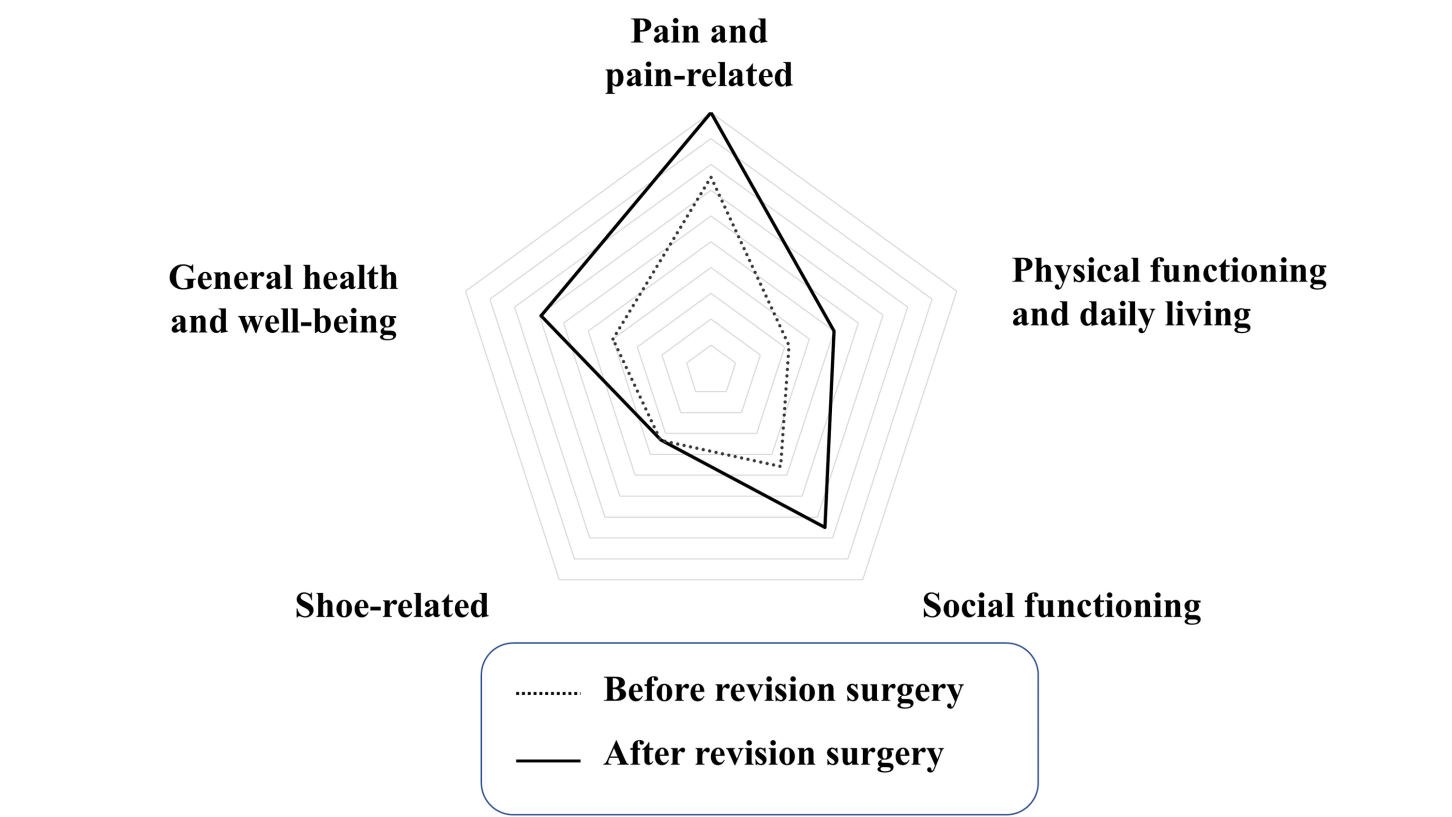
Improvement in the SAFE-Q score was evident at 4 years after the revision surgery except for shoe-related issues. SAFE-Q: self-administered foot evaluation questionnaire

The patient walked with a patellar tendon-bearing brace for two weeks post-surgery, starting with partial weight bearing from six weeks post-surgery and reaching full weight bearing at 12 weeks. One year after the surgery, bone fusion was complete, the flat foot was plantigrade, and stable standing and walking were possible. Four years after the surgery, the range of motion at the ankle joint was maintained at 10º of dorsiflexion and 40º of plantar flexion. By this time, although some remaining β-tricalcium phosphate could be seen on an anteroposterior plain radiograph, bone fusion was complete (Figures 6, 7). The tibial articular surface improved to 90º, the talar tilt angle to 0º, the calcaneal pitch to 22º, and the Meary’s angle to −14º. Instability when weight bearing disappeared and she was able to walk continuously for 400 m. Her preoperative JSSF (Japanese Society for Surgery of the Foot) [2, 3] score of 43 points increased to 88 points, with overall improvement in pain, function, and alignment (Figure 8, Appendix 1). Her scores on the self-administered foot evaluation questionnaire (SAFE-Q) [4-6], which is a patient-based evaluation, did not indicate a significant change over time at one year after surgery. However, two years after the surgery, improvement was seen in the pain and pain-related, physical functioning and daily living, and social functioning items of the SAFE-Q. By four years, the results for general health and well-being had also improved significantly (Figure 9, Appendix 2). Her shoe-related scores remained the same as before the surgery because of the foot drop. Her gait was improved by the use of a brace to support the foot drop. The patient’s overall satisfaction was her surgery was excellent.

## Discussion

Triple arthrodesis is often performed for clubfoot and pes cavus associated with paralytic disease. However, problems can occur after this type of surgery, including osteoarthritis and malalignment. Reports indicate that 39%-77% of cases have osteoarthritis of the adjacent joints in the long term [7]. However, functional problems are rare [7]. In one report, malalignment was valgus-type in 33% of cases and varus-type in 22% [8]. Additional surgery may be required if the patient has symptoms or if the problem worsens over time [1]. If varus-type malalignment or Achilles tendon contracture remains after surgery, varus stress is applied to the ankle joint because of loss of movement at the subtalar joint. As a result, supination of the foot is common with lateral ligament injury in the ankle in adults and ball-and-socket deformity may develop in the ankle joint while in children. In a ball-and-socket ankle, movement of the subtalar joint is restricted before the end of the growth period. Therefore, the ankle joint grows as a compensatory mechanism [9], and the articular surface of the talus becomes spherical [10]. In our case, in addition to a slight ball-and-socket ankle, varus stress was repeatedly applied to the ankle joint when weight-bearing, leading to a gradual increase in lateral ligament insufficiency and the ankle joint being subjected to significant varus stress.

If a varus deformity occurs at the ankle joint after triple arthrodesis, an ankle arthrodesis, and calcaneal osteotomy (such as the Dwyer procedure) are performed to correct the hindfoot [1]. However, we could find no detailed examination of this approach in the literature. In this case, the range of motion at the ankle joint was maintained and the arthropathy had not progressed. Therefore, joint-preserving surgery was considered. However, there was also instability (incompatibility with the ankle mortise) as a result of plafond tilt and ankle deformity, and there was a possibility that the problem could not be improved by calcaneal osteotomy and lateral ligament reconstruction for the ankle alone. The postoperative course after the previous surgery was complicated by infection. We considered that leveling the plafond and stabilizing the ankle joint by including a distal tibial oblique osteotomy was appropriate. Kim et al. correlated the postoperative talar tilt angle with clinical outcomes and recommended a dilatation angle of 88º, which is the average Japanese talar tilt angle [11]. We also used this as a reference, and the plafond and talus were leveled with respect to the axis of the tibia, thereby improving compatibility with the ankle mortise [12]. Therefore, the stability of the ankle joint was improved. Dwyer calcaneal osteotomy is also useful for hindfoot varus. However, the procedure is complicated. If the deformity is not severe, a lateral displacement calcaneal osteotomy is sufficient and the procedure is straightforward. For these reasons, we chose that procedure for this complicated case.

Intraoperative challenges include the limited mobility of the calcaneus, which can only be shifted up to 10 mm, and the numerous procedural steps required to complete the surgery. To date, there has been no precedent in the literature since Coughlin et al. described salvage surgery that combines hindfoot flatfoot treatment with simultaneous varus correction for malalignment following triple-joint replacement surgery [1]. Postoperatively, varus deformity did not recur, and a stable flatfoot was achieved. The outcomes were favorable, including increased walking endurance and overall patient satisfaction.

## Conclusions

Triple arthrodesis is a common procedure to correct deformities such as clubfoot or pes cavus in paralyzing conditions, but it can lead to complications such as osteoarthritis and malalignment over time. These problems may require additional corrective surgery. In cases of varus deformity after triple arthrodesis, joint-preserving options such as distal tibial oblique osteotomy and calcaneal osteotomy can stabilize the ankle and improve alignment. Although this approach proved effective in this case, a stable, functional foot with improved gait endurance was achieved. The combination of plafond leveling and hindfoot correction offers a promising solution. 

## Figures and Tables

**Figure 10:**
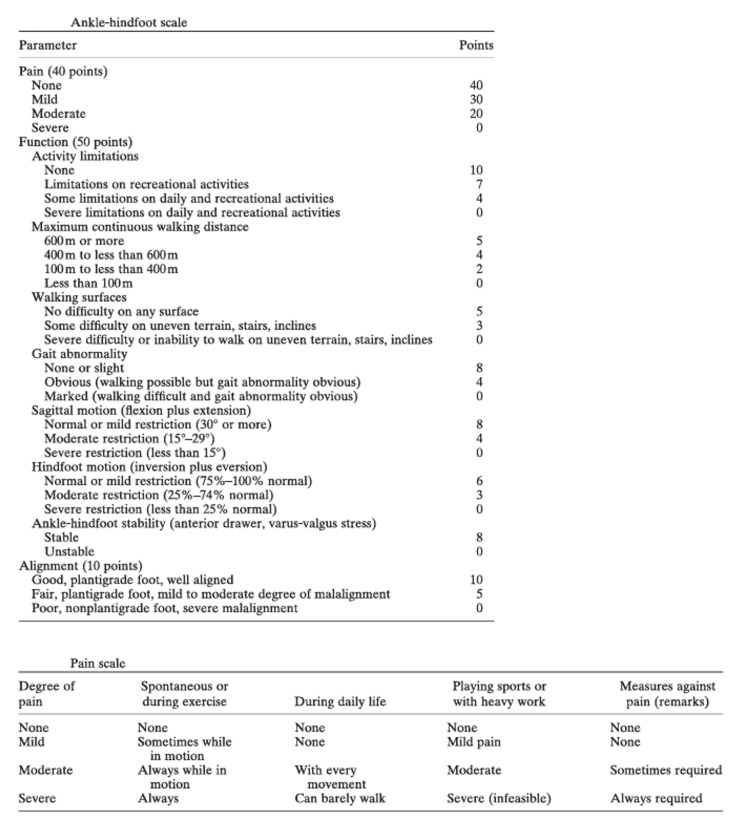
The questionnaire for Japanese Society for Surgery of the Foot (JSSF) score.

**Figure 11:**
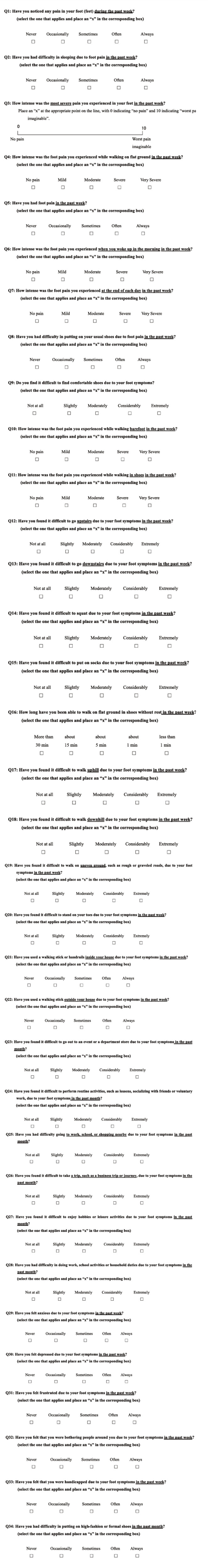
The questionnaire for the self-administered foot evaluation questionnaire (SAFE-Q)

## Appendices

Appendix one 

Appendix two 
